# Exploring COVID-19 patients’ experiences of psychological distress during the disease course: a qualitative study

**DOI:** 10.1186/s12888-021-03626-z

**Published:** 2021-12-11

**Authors:** Tahereh Toulabi, Fatemeh Jafari Pour, Atefeh Veiskramian, Heshmatolah Heydari

**Affiliations:** 1 Department of Critical Care Nursing, School of Nursing and Midwifery, Lorestan University of Medical Sciences, Khorramabad, Iran; 2 Department of Nursing, Behbahan Faculty of Medical Sciences, Behbahan, Iran; 3 Social Determinates of Health Research Center, Lorestan University of Medical Sciences, Khorramabad, Iran; 4 French Institute of Research and High Education (IFRES-INT), Paris, France

**Keywords:** COVID-19, Patients, Psychological distress, Qualitative study

## Abstract

**Background:**

The Coronavirus Disease 2019 (COVID-19) is an emerging disease with many unknown clinical and therapeutic dimensions. Patients with COVID-19 experience a variety of psychological problems during the disease. Understanding patients’ mental condition and their distress during the disease is the first step to help these patients. So, the aim of this study was to explain COVID-19 patients’ experiences of psychological distress during the disease course.

**Methods:**

The present qualitative research was conducted in Iran from April 2020 to April 2021 using the conventional content analysis method. The participants included patients with COVID-19, selected by the purposeful sampling method. Data was collected through 34 telephone and in-person interviews and analyzed based on the method proposed by Lundman and Graneheim.

**Results:**

Qualitative data analysis led to the emergence of sources of psychological distress as the main theme as well as seven categories and seven sub-categories. The categories were the disease’s nature (the subcategories of disease’s unknown dimensions, and disease severity), the anxiety caused by preventive behaviors (the subcategories of quarantine, worry about transmitting the infection to others and obsessive thoughts related to disinfection measures), the inefficient management by the health system (the subcategories of poor health care condition and lack of spiritual care), death anxiety, stigma, anxiety after recovery, and sleep pattern disturbance.

**Conclusion:**

Patients with COVID-19 experience great psychological distress during the acute phase of the disease or even long after recovery. It is suggested that psychological and spiritual counseling, as a key element of treatment and support for these patients, is provided to patients in the acute phase of the disease, as well as after recovery. National and local media should boost awareness about the disease as a dangerous yet preventable and curable infectious disease. People should follow health instructions and leave their seeing the disease as a taboo.

**Trial registration number:**

Not applicable.

## Background

The Coronavirus Disease 2019 (COVID-19), as an emerging infectious disease, was first reported on December 31, 2019, in Wuhan, China [1]. The World Health Organization (WHO) declared the disease a pandemic on March 11, 2020, due to its rapid spread, involving most countries across the world [2], affecting the health, social, and economic sectors. Due to its high transmission and mortality rates, the pandemic was declared as a global public health emergency [3]. The disease is highly contagious and is transmitted among humans via contaminated respiratory droplets and surfaces, as well as close contact [4]. Until April 14, 2021, the number of COVID-19 cases had stood at 138,344,571, and the number of deaths had reached 2,977,136 worldwide [5]. About 20% of patients may experience severe symptoms, requiring oxygen therapy or other hospital-based interventions, and only 5% of patients may require hospitalization in the intensive care units [6]. The mortality rate of COVID-19 is estimated between 1 and 5%, but it varies depending on the patient’s age and the presence of comorbidities [6].

Similar to other parts of the world, Iran has been suffering from the COVID-19 disease for over a year and has experienced four waves of the disease so far. Until April 14, 2021, the number of patients with COVID-19 in the country had reached 2,143,794 cases, claiming 65,359 lives [5].

The results of studies on patients with COVID-19 indicate that, in addition to physical symptoms, they may experience various mental health problems [7–10]. The development of psychological distress was also reported during/after the SARS-COV crisis [11]. People may experience psychotic distresses even after recovering from COVID-19 [12, 13].

Psychological distress can play an important role in determining the severity and recurrence of acute respiratory infections [14]. Stressful life events and perceived stress during life can activate the hypothalamic-pituitary-adrenal axis, as well as the autonomic nervous system [15]. Elevated levels of stress hormones disrupt inflammatory responses by suppressing the humoral and cellular immune systems and altering the balance of proinflammatory cytokines [16]. Recent studies have shown changes in the immune function of COVID-19 patients [17]. Nevertheless, less attention has been paid to these patients’ psychological needs and the necessity of providing psychological support to them, as a neglected link in this area [18]. Therefore, it is necessary besides paying attention to patients’ physical problems to consider their psychological distress as well.

These problems should be divulged and explained by patients themselves in order to identify the type of psychological problems and their root causes and also explain the signs and condition of patients during the disease. A complete understanding of these patients’ psychological and mental problems can help take appropriate decisions to manage their psychological distress and provide them with appropriate care services.

One of the proper ways to understand patients’ psychological distresses is to interview them, which is achievable through qualitative interviews. Given the expertise of the authors in conducting qualitative research and their engagement in providing care to COVID-19 patients, this study was performed to explore COVID-19 patients’ experiences of psychological distress during the disease course.

## Materials and methods

This qualitative study was conducted according to the conventional content analysis approach.

### Participants

Study participants included the patients contracting the COVID-19 disease amid the pandemic, who were recruited by the purposeful sampling method. Inclusion criteria were the confirmed diagnosis of COVID-19 disease based on RT-PCR, passing at least 2 weeks from the diagnosis, experiencing the clinical symptoms of the COVID-19 disease, referral to health care centers and receiving outpatient or inpatient care, and willingness to participate in the study. Exclusion criteria included inability or unwillingness to participate in the study.

### Data collection

Considering the immediate need for the data to improve the patient care process and due to limitations in conducting face-to-face interviews during different waves of the disease in Iran (four waves), the data was collected via semi-structured in-depth telephone or face-to-face interviews from April 1, 2020 to the end of March 2021. Patients with a positive PCR test and a history of hospitalization were identified by referring to the infection control offices of hospitals. The outpatients seeking health services were also identified by visiting clinics and physician offices. By referring to the staff providing health care to these patients (hospitals, clinics, or doctor offices), the patients who had rich information relevant to the purpose of the study were purposefully selected. After the patients were approved by the doctors and nurses who provided health care to them, they were contacted via phone calls, and if they were found to be eligible and available to participate in the study, the time and place of the interview were scheduled. The first, second, and third authors conducted the interviews and then transcribed them. After explaining the goals of the study and obtaining informed consent, the duration of the interview was determined based on the desire, patience, and richness of the participant’s experiences. Face-to-face interviews were conducted according to the participants’ preferences in either medical centers, patients’ homes, or a quiet and comfortable place. All the interviews were recorded using an electronic device. The main question of the study was: “Could you please talk about your COVID-19 experience?” and “Could you please share with us your psychological status during the disease?”. Then based on the participant’s responses, more detailed questions such as “Could you please share an experience?”, “What do you mean?”, and “Could you please explain more about this?” were asked.

### Data analysis

All authors participated in data analysis. The initial analysis was conducted by the corresponding author, and then other authors took part in correcting, entitling, and finalizing categories and subcategories. A final consensus was made on the categories and subcategories. Data analysis was conducted based on the Lundman and Graneheim approach [19], who suggested five steps for data analysis as follows: 1- transcribing the entire interview immediately after its completion, 2- reading the whole text of the interview to obtain a general understanding of its content, 3- determining the meaning units and initial codes, 4- classifying similar initial codes under broader categories, and 5- determining hidden concepts within the data.

Accordingly, data analysis was conducted inductively. The data gathered was analyzed simultaneously with the interviews. For this, the interviews were immediately transcribed verbatim, and the written data were read for several times to arrive at a general understanding of their content. The initial codes were extracted from the data. A thorough interview transcript was regarded as the unit of analysis, and meaning units included words, sentences, and paragraphs. The initial codes were compared with each other to merge related ones and form categories and subcategories based on their similarities and differences. The categories were also compared with each other and grouped into higher-level main theme.

### Trustworthiness

The accuracy and reliability of the data were determined based on the method proposed by Lincoln and Guba [20]. The criteria used included credibility, transferability, and dependability.

The prolonged (i.e., 1 year) engagement of authors with the data helped to achieve credibility. For further confirming data credibility, a peer-debriefing session was held with two faculty members who were experts in qualitative research methods and agreed on the extracted codes and their classification. Member-checking was done after extracting the initial codes, and the accuracy of the extracted codes was confirmed according to the participants’ viewpoints. In the case of any inconsistency with the participants’ comments, the corrections required were considered. The participants had the maximum diversity for age, sex, and level of education, which could have helped boosting data credibility.

For data transparency, the authors tried to write the manuscript with sufficient details. For dependability, the processes of studying and interpreting the data were supervised by a scientific committee.

### Ethical considerations

All methods were performed in accordance with the relevant guidelines and regulations by in the declaration of Helsinki (ethics approval and consent to participate). The aims and methods of the study were explained for all participants, and necessary assurance was given to them for the anonymity and confidentiality of their information and audio files. Informed consent (with explaining the goals and methods of the study) was obtained from participants. The interviewers were reading text of informed consent form verbatim for illiterate participants and obtained informed consent of them. And also, informed consent was obtained from parent or legal guardian for illiterate participants. The participants had the right to withdraw during the study or at any other time. The Ethics Committee of Lorestan University of Medical Sciences approved the study protocol (ethical code: IR.LUMS.REC.1399.018).

## Results

Overall, 34 patients with COVID-19, including 17 (50%) females and 17 (50%) males, participated in this study. The participants’ mean age was 49.79 ± 15.03 years, and 24 of them were married. The data were collected through 14 face-to-face interviews and 20 phone interviews (Table 1).

**Table 1:** Demographic Characteristics of Participants

Participant No.	Sex	Age (Year)	Marriage status	Education level	Job	Type of Interview
1	Woman	22	Married	Undergraduate student	Student	Telephone
2	Woman	66	Married	Undergraduate degree	Retired	Telephone
3	Man	57	Married	Bachelor degree	Retired	Telephone
4	Man	55	Married	Undergraduate degree	Retired	Telephone
5	Woman	47	Married	High school	Housewife	Telephone
6	Woman	60	Married	Illiterate	Housewife	Telephone
7	Man	50	Married	Undergraduate degree	Employee	Telephone
8	Man	25	Single	Post graduate	Employee	Telephone
9	Man	52	Single	Undergraduate degree	Employee	Telephone
10	Man	66	Married	Diploma	Retired	Telephone
11	Woman	72	Married	Illiterate	Housewife	Telephone
12	Man	74	Married	Illiterate	Retired	Telephone
13	Man	70	Married	Undergraduate degree	Retired	Telephone
14	Woman	66	Married	Illiterate	Housewife	Telephone
15	Man	47	Single	Illiterate	Retired	Telephone
16	Man	56	Married	Post graduate	Employee	Telephone
17	Man	28	Single	High school	Unemployed	Telephone
18	Man	51	Married	Bachelor degree	Self-Employment	Telephone
19	Man	27	Married	High school	Self-Employment	Telephone
20	Man	42	Single	Bachelor degree	Employee	Telephone
21	Woman	38	Single	Bachelor degree	Employee	Face to face
22	Woman	33	Single	Post graduate	Employee	Face to face
23	Woman	72	Married	Illiterate	Housewife	Face to face
24	Man	57	Married	Diploma	Retired	Face to face
25	Man	43	Married	Bachelor degree	Employee	Face to face
26	Man	52	Married	Bachelor degree	Employee	Face to face
27	Woman	61	Married	Illiterate	Housewife	Face to face
28	Woman	26	Single	Bachelor degree	Employee	Face to face
29	Woman	51	Married	Reading and writing	Housewife	Face to face
30	Woman	26	Single	Bachelor degree	Employee	Face to face
31	Woman	51	Married	Bachelor degree	Employee	Face to face
32	Woman	55	Married	Post graduate	Faculty member	Face to face
33	Woman	38	Single	Bachelor degree	Employee	Face to face
34	Woman	57	Married	Illiterate	Housewife	Face to face

Qualitative data analysis led to the emergence of one theme, seven categories, and seven sub-categories (Table 2).

**Table 2:** The Main Themes, Categories, and Subcategories Extracted from the Data

Themes	Categories	Sub-categories
Sources of psychological distress	The disease’s nature	The disease’s unknown dimensions
		The disease severity
	Anxiety related to preventive behaviors	Quarantine
		Worry about transmitting the infection to others
		Obsessive thoughts related to disinfection measures
	Inefficient management by the health system	Poor health care condition
		Lack of spiritual care
	Death anxiety	
	Stigma	
	Anxiety after recovery	
	Sleep pattern disturbance	

### Sources of psychological distress

Our data shows that the COVID-19 causative agent is a virus with a high mutation rate, which changes itself every day and infects many while killing a considerable number of people. Therefore, patients have to adhere to health and quarantine protocols for their own and others’ safety. On the other hand, the health system has encountered problems providing comprehensive patient care due to an overwhelming number of infections and shortages in protective equipment and improper infrastructure. All these have caused psychological and mental distress for patients.

Under this main theme, seven categories and seven sub-categories were located, including disease’s nature (the subcategories of disease’s unknown dimensions, and disease severity), anxiety related to preventive behaviors (the subcategories of quarantine, worry about transmitting the infection to others and obsessive thoughts related to disinfection measures), inefficient management by the health system (the subcategories of poor health care condition and lack of spiritual care), death anxiety, stigma, anxiety after recovery, and sleep pattern disturbance.

#### The Disease’s nature

The condition created by the COVID-19 in the community has brought fear and distress among patients. In fact, the lack of preventive measures and definitive treatments, the increasing rate of the infection across the community, numerous mutations, the high mortality rate, and also a bad view towards the patients by the society have caused distress among the patients. Within this category, there were the subcategories of the disease’s unknown dimensions, and disease severity.

##### The Disease’s unknown dimensions

The COVID-19 is an emerging disease whose many dimensions in terms of prevention and treatment are unknown. Data analysis showed that the disease being unknown, the unpredictability of the patient’s condition, lack of definitive treatments, the growing trend of the disease’s incidence, as well as the occurrence of frequent mutations were sources of fear and anxiety in patients. One of the participants mentioned: “…it was an unpleasant and scary experience. It was something unknown…” (20). Another participant elaborated about the unknown nature of the virus: “… the virus is still unknown …” (22). Explaining the unpredictability of the patient’s outcome, one of the participants noted: “… I was anxious about not knowing what would happen … “(5). Another participant, citing the uncertainty about his fate, said: “…It is not clear whether you regain your health or not …” (24). Another participant explained about being unaware about returning to normal life: “… I do not know exactly what I should do about marital relations…” (1). Data analysis showed that the growing trend of the disease’s incidence, as well as upward trends in mortality rates could cause stress and anxiety in patients. A participant mentioned: “…when I contracted the disease… hearing the high mortality rate made me stressed and aggravated … “(5).

##### The Disease’s severity

Data analysis showed that the high mortality rate of the disease caused anxiety and stress in patients. Regarding this issue, one of the patients elaborated on the fear of the disease’s fatality: “… I assumed that if everyone gets infected, he would die … I would imagine death in front of my own eyes …” (2). Another participant said: “…regarding the invasion of COVID to my body and the inflicted pressure, I must say… you feel it is the end of your life and you cannot return …” (24). This participant continues talking about the disease’s fatality: “…a difficult situation between life and death … there is no hope…even the people who had good conditions and talked to you…. Later on, you realize that they have died…” (24).

#### Anxiety related to preventive behaviors

Data analysis showed that the observance of preventive behaviors by patients with COVID-19 could cause them psychological distress. During the disease, patients should live away from others for a while to stop disease propagation. The observance of health protocols and preventive behaviors by patients and their ignorance by the community can have adverse effects on patients’ psychological well-being. Within this category, there were three subcategories of quarantine, worry about transmitting the infection to others, and obsessive thoughts related to disinfection measures.

##### Quarantine

Patients are psychologically affected during quarantine and loneliness and become irritable and vulnerable. They feel themselves far from the family and see quarantine as the end of life. One of the participants described her bad feeling during quarantine: “…At home and in quarantine, I felt it was the end of my life, and that I was dying … I felt like an extra being… “(1). Another participant mentioned: “ … It was a very bad feeling. I was scared and crying most times … “(32); another participant noted: “…the feeling of loneliness was agonizing and annoying …” (29). One participant, about the bad feeling of being far from the family, stated: “… what I hated was that I was away from the family .... I have not seen my mother for 40 days …” (2). Data analysis also showed that the time passed in quarantine to the further development of psychological disorders. In this regard, one patient expressed: “…my brother developed depression… and now he does not talk to anyone … “(17).

##### Worry about transmitting the infection to others

Data analysis indicated that among the reasons for patients’ fear and anxiety were their concerns about infecting their families and other members of the community with the COVID-19 causative virus. In this regard, one of the patients said: “… after I realized that my corona test was positive, my greatest anxiety was because of infecting my wife and children…” (31). Another participant, addressing the influence of the fear of infecting others on routine life, mentioned:“.... For example, there was anxiety about going shopping, that if I go, the seller would be affected. .... and if I disclose that I am positive to get the seller alerted, you would create anxiety in that person .... “(28). Also data analysis showed that one of the sources of stress and anxiety in patients was the public not adhering to health guidelines. In this regard, one of the participants noted: “… Yesterday, I had to go out … and I encountered an unpleasant scene… everywhere was crowded, and many did not use face masks and gloves and ignored social distancing …” (15).

##### Obsessive thoughts related to disinfection

Data analysis showed that patients had developed obsessive thoughts due to excessive washing of hands, objects, and other materials, as well as excessive use of disinfectants, causing them disturbance and anxiety. On this issue, one of the participants stated: “.... I have not taken a taxi for a long time because I am very afraid of touching money. The obsession about spraying alcohol on everyone and everything…” (20). Another participant said:“…During this time, I considered everything and everyone contaminated and wore gloves outside the home ....” (14).

#### Inefficient management by the health system

Data analysis showed that patients with the COVID-19 disease need to use the capacity of health care centers. The plans, structure, manpower, as well as equipment of health care centers can affect patients’ psychological well-being. In this category, two subcategories, including poor care conditions, lack of spiritual care, were identified.

##### Poor health care conditions

Data analysis showed that the patient’s care condition can be a source of either comfort or distress. The prevailing atmosphere, the behavior of health staff, number of hospitalized patients in the ward, observing the death of patients, as well as support and encouragement by health staff can influence patients’ psychological conditions. In this regard, one of the participants mentioned the bad behavior of health staff as a stressor and added: “.... I was upset with the behaviors of the hospital’s staff and manager...” (21). Another participant perceived the poor care condition as a source of fear and anxiety: “…the poor care condition deteriorated my spirit…” (2). Data analysis showed that one of the factors that can affect patients’ psychological state was the discipline and cleaning of the hospital environment. In this regard, one of the participants explained about the lack of cleaning of the ward: “… toilets were dirty due to the ignorance of some patients, especially elder ones…” (20). One participant noted: “…pillows and sheets were hardly available. There were patients who were hospitalized for 14 days but had not yet taken a shower ...” (18). Another participant addressed the lack of manpower and its impacts on patients’ spirits: “…nurses could not provide all services to patients because of a shortage in manpower …” (5). Data analysis showed that patients were afraid of referring to hospitals due to the crowded medical centers. One of the participants mentioned the case of his father: “…Unfortunately, my father refused to go to the hospital due to bad news about them, overcrowding, the high mortality rate, conflicts, etc..... he refused to go to the hospital …” (22).

##### The lack of spiritual care

One of patients’ care needs is paying attention to the spiritual dimension, which has been neglected in patients with COVID-19, and this can be a source of fear and anxiety. In this regard, one of the participants highlighted inadequate facilities for fulfilling patients’ spiritual needs: “…there was no place for praying in the hospital …” (3). Also, data analysis revealed patients’ needs to talk with religious experts about death and afterlife; one participant elaborated: “…I wanted to talk to someone who was fully aware of afterlife…” (33).

#### Death anxiety

Data analysis revealed patients were stressed and anxious of death due to their attachments as well as the unknown nature of death. Some patients were afraid of death because of the loneliness of their families and relatives. In this regard, one of the participants expressed his concerns about leaving his wife alone”: “… I was worried about what would happen to my wife after my death …” (13). Another participant mentioned: “… I am too attached to my little girl and the thought of how she would be after my death made me feel bad and worried … “(6). The patients were horrified by the death due to COVID-19. One of the participants said: “…I think death due to COVID is a strange phenomenon… is death in exile... “(5); another participant about his anxiety stated: “… the shortness of breath made me feel inside a grave, and the image I had about the funeral of COVID patients… being placed into graves by a shovel … pouring lime on them… made me very sad…” (3).

#### Stigma

The COVID-19 disease is recognized as a social stigma, and patients are reluctant that those around them know about their illness, exaggerating their anxiety and distress. During the course of the disease, patients isolate themselves from the community, and even after recovery, they fear entering the community. On the other hand, some people in the community avoid recovered patients, leading to stress and anxiety in patients. In this regard, one of the participants mentioned: “… COVID has become a disgrace, and whoever has it tries to hide it …” (20). Addressing the bad behavior of those around him, a participant mentioned:“…Sometimes that relatives called …they behaved as if you were impure…” (4). Another participant shared his concerns about neighbors’ becoming aware of his disease: “…this disease has a bad image in the society .... even when health experts came for disinfection two or three times …I told them not to come anymore because it would be a bad image if relatives or neighbors find out…” (1).

#### Anxiety after recovery

Data analysis showed that some patients had the stress of being reinfected after recovery. One of the participants mentioned: “.... I am afraid that I may be infected again, and the fact that I am not immune to the disease…. even though I have started my work again, I am always worried about being re-infected … “(17). Another participant noted: “… I am under a lot of stress because of these symptoms and the disease’s recurrence…” (3). Another patient said:“ … I am afraid of disease recurrence now…” (14). Another participant mentioned his concerns about the future of the disease: “… this stress continues .... the virus is still unknown, and people may get re-infected even after recovery… or a complication may persist…” (22). Data analysis also showed that patients may live with the symptoms of the disease for a long time. The participants mentioned that living with the symptoms of this disease could cause them anxiety. Regarding this, one of the participants mentioned: “.... I am still suffering from the disease’s symptoms … I did not completely recover and become really irritable …” (32).

#### Sleep pattern disturbance

Data analysis indicated that patients experienced sleeplessness at night due to various problems and the symptoms of the disease. The participants’ experiences indicated that most of the disease’s symptoms, such as systemic, respiratory, and gastrointestinal complications, as well as headache, and anxiety were exaggerated at night, interfering with sleep and causing the patient to remain up all night. During the day; however, symptoms improved, allowing patients to sleep. According to one of the participants: “…I was very restless at night… felt heavy in my chest and could not sleep …” (7). Another participant said: “…I had pain, fever, and chills at night….my night sleep was disturbed… and I was sleeping during the day …” (3). Another participant cited: “… respiratory distress was exaggerating at night, disturbing sleep… “(4). Data analysis also showed that COVID-19 patients may experience nightmares due to severe pain and discomfort. The participants noted that tthoughts about losing some body parts and reviewing unpleasant childhood memories may be seen. In this regard, one of the participants mentioned: “…when I was sleeping on the hospital bed, I felt like I only had a brain and a heart and none of the other organs of my body…” (24), and continued: “…All my life, from childhood to the present, passed before my eyes ... these feelings came to me like dreams or as an external existence, it was like a movie playing in front of my eyes…” (24). Data analysis showed that wearing face masks constantly was annoying for patients, causing them discomfort and anxiety. One of the participants cited the agony caused by wearing a face mask “.... I was feeling suffocated. Wearing a face mask at home is nauseating …” (8).

## Discussion

Data analysis indicated that patients with COVID-19 experienced many psychological distresses during the acute phase of the disease and even long after recovery. Various factors such as the nature of the disease, anxiety related to preventive behaviors, inefficient management by the health system, death anxiety, stigma, anxiety after recovery, and sleep pattern disturbance cause stress and anxiety in patients. In line with the findings of this study, many studies have highlighted the presence of psychological distresses among patients with COVID-19 [18, 21, 22], many of which are encountered during hospitalization [23].

According to our data, the nature of the disease was a source of distress in COVID-19 patients. The lack of therapeutic measures, the increasing trend of the infection at the community level, the occurrence of numerous mutations, and the high mortality rate are assumed to cause stress in patients. In line with our findings, the fear and anxiety related to the unknown dimensions of the disease were reported in patients with COVID-19 in another study [18]. On the other hand, fear of unknown entities, as a source of anxiety, can affect the immune system [24, 25]. Besides the efforts of the international community to identify the disease’s various dimensions, authorities should focus on the known aspects of the disease and implement special measures for public vaccination, especially for the most vulnerable individuals.

Our findings showed that COVID-19 patients developed anxiety due to preventive behaviors. Quarantine and loneliness were sources of distress in COVID-19 patients. In this regard, the findings of a study showed that married patients with COVID-19 had experienced more psychological problems [23], which could be attributed to the unstable condition of these patients [26] or the sense of responsibility of married people to their families. Officials can use the capacity of the national TV to broadcast entertaining programs for quarantined people during the acute phase of the disease to alleviate their anxiety. Patients can also entertain themselves by watching movies, reading books, and doing fun and interesting games. Using the capacity of social media, as well as phone calls from relatives and friends can also help patients to feel less lonely. Another issue that can concern patients is the attitude of the general population towards preventive guidelines. So, officials with the help of national and local media should inform the public about the disease, recognize supportive and psychological needs of COVID-19 patients, and follow health instructions.

Our findings showed that COVID-19 patients had experienced obsessive thoughts. Consistent with this finding, other studies confirmed the occurrence of obsessive thoughts and post-traumatic stress in patients with COVID-19 [27, 28]. Therefore, it seems necessary to identify the patients who are at risk of obsessive problems using appropriate tools and to consider appropriate supportive programs for them based on standard guidelines [29].

Data analysis showed that one of the sources of anxiety in COVID-19 patients was the disease being recognized as a stigma. In line with the findings of this study, the results of another study showed that stigma can affect COVID-19 patients’ lives socially, personally, and economically [30]. Social stigma delays treatments and facilitates disease progression and propagation in the society [31]. By broadcasting various programs, the national television should boost social culture in this regard and inform the public about the curability of the disease.

Data analysis showed that the poor management of the pandemic by the health system induced anxiety in COVID-19 patients due to inappropriate environmental conditions. Patients with COVID-19 had been admitted in crowded rooms alongside other patients with critical conditions.

Consistent with the findings of this study, the results of another study showed that witnessing the death of critically ill patients was one of the sources of stress in the patients admitted to the hospital [32]. Hospitals’ authorities can categorize patients based on the disease’s severity, host them in different wards, and provide them with health services in a comfortable and calm environment.

According to our findings, COVID-19 patients faced some challenges during their treatment process. In line, another study declared that the lack of manpower, insufficient equipment, and a shortage in hospital beds could result in stress and anxiety [33]. Managing available resources, paying attention to positive international experiences, and using the potential of social capital can be effective steps in this area [34]. So, it seems necessary that the structure and processes of care provision to COVID-19 patients be re-designed to be able to deliver quality services to patients.

Data analysis highlighted the lack of spiritual care and death anxiety among the important problems of patients with COVID-19. In line with these findings, another study showed that death anxiety in patients with COVID-19 can occur due to social isolation, restrictions in visits, and distance from the family [32]. Death anxiety could also be due to the unknown afterlife world and the bad image of the burials of COVID-19 patients’ bodies. It seems that psychological and spiritual counseling to patients with COVID-19 in order to explain the life process and the fact that death is a part of life can help to reduce anxiety in patients to some extent.

Our data showed that patients may experience post-recovery anxiety. In parallel, other studies showed that more than half of patients with SARS-COV infection still suffered from psychological problems even months after recovery [35, 36]. Considering that the COVID-19 disease in many aspects is more severe than SARS [37], it seems that more measures should be implemented to prevent its psychological consequences in affected people.

Sleep pattern disturbance was noted as another problem of COVID-19 patients. In line with this, sleep disorders were reported in another study [38]. Sleep plays an important role in regulating the cellular and humoral immunities, and insufficient sleep can weaken immune responses to the virus [39]. Our findings showed that physical problems may be in part involved in patients’ sleep disturbance, which may be resolved by using appropriate drugs [40]. There are various guidelines to help manage sleep disturbance during epidemics, which can be employed by authorities to improve the sleep quality of patients [41, 42]. To achieve this goal, patients should receive care in quiet places, and proper disciplines should be implemented in wards to ensure greater comfort for patients.

One of the limitations of this study was that due to the restrictions caused by the pandemic, some of the interviews were conducted by phone calls, which could have affected data richness. By conducting in-person interviews as much as possible, we tried to overcome this shortcoming. Considering that the interviews were conducted after the recovery of patients (i.e., when they had been discharged from the hospital), finding the key informant was somehow difficult. So, we tried to minimize the impact of this item by referring to the health care providers who knew the patients. Because of the risk of virus transmission during the interview, health protocols were followed as much as possible.

## Conclusion

Patients with COVID-19 experience a variety of psychological distress during the acute phase of the disease or even long after recovery. Various factors, including the nature of the disease, anxiety related to preventive behaviors, insufficient management by the health system, death anxiety, stigma, anxiety after recovery, and sleep pattern disturbance can trigger psychological distresses in COVID-19 patients. The national and local media can play an essential role amid this critical situation. They can inform people and boost social culture about the disease, highlighting that it is a dangerous, yet preventable and treatable, infectious disease, so they follow health instructions. In addition, the belief of the disease being a taboo can be alleviated. Psychological and spiritual counseling, as a key component of support programs for COVID-19 patients, should be considered during the acute phase of the disease and after recovery.

## Data Availability

The datasets generated and/or analyzed during the current study are available on request from the corresponding author.

## Abbreviations

COVID-19: Coronavirus disease- 2019
MERS-COV: Middle East Respiratory Syndrome coronavirus
SARS-COV: Severe acute respiratory syndrome coronavirus
WHO: World Health Organization
